# Performance Benchmarks for Scholarly Metrics Associated with Fisheries and Wildlife Faculty

**DOI:** 10.1371/journal.pone.0155097

**Published:** 2016-05-06

**Authors:** Robert K. Swihart, Mekala Sundaram, Tomas O. Höök, J. Andrew DeWoody, Kenneth F. Kellner

**Affiliations:** 1 Department of Forestry and Natural Resources, Purdue University, West Lafayette, Indiana, United States of America; 2 Illinois-Indiana Sea Grant Program, Purdue University, West Lafayette, Indiana, United States of America; Hellenic Centre for Marine Research, GREECE

## Abstract

Research productivity and impact are often considered in professional evaluations of academics, and performance metrics based on publications and citations increasingly are used in such evaluations. To promote evidence-based and informed use of these metrics, we collected publication and citation data for 437 tenure-track faculty members at 33 research-extensive universities in the United States belonging to the National Association of University Fisheries and Wildlife Programs. For each faculty member, we computed 8 commonly used performance metrics based on numbers of publications and citations, and recorded covariates including academic age (time since Ph.D.), sex, percentage of appointment devoted to research, and the sub-disciplinary research focus. Standardized deviance residuals from regression models were used to compare faculty after accounting for variation in performance due to these covariates. We also aggregated residuals to enable comparison across universities. Finally, we tested for temporal trends in citation practices to assess whether the “law of constant ratios”, used to enable comparison of performance metrics between disciplines that differ in citation and publication practices, applied to fisheries and wildlife sub-disciplines when mapped to Web of Science Journal Citation Report categories. Our regression models reduced deviance by ¼ to ½. Standardized residuals for each faculty member, when combined across metrics as a simple average or weighted via factor analysis, produced similar results in terms of performance based on percentile rankings. Significant variation was observed in scholarly performance across universities, after accounting for the influence of covariates. In contrast to findings for other disciplines, normalized citation ratios for fisheries and wildlife sub-disciplines increased across years. Increases were comparable for all sub-disciplines except ecology. We discuss the advantages and limitations of our methods, illustrate their use when applied to new data, and suggest future improvements. Our benchmarking approach may provide a useful tool to augment detailed, qualitative assessment of performance.

## Introduction

University administrators seek evidence of scholarly productivity and impact when making decisions on, among other things, faculty hiring, salary increases, tenure, and promotion. Ideally, evidence is multi-faceted and includes data collected objectively from multiple sources [1] as well as insights provided by peers or client groups. Evidence of productivity and impact in teaching, for example, may include student credit hours generated, number of courses taught, student and peer evaluations, accounts of mentoring, curriculum development and innovation, funds obtained in support of teaching/learning, and publication of teaching practice or scholarship.

In research, evidence of scholarship is largely (though not exclusively) dependent on a faculty member’s publication and citation record. To the extent that science operates on an “economy of reputation” [2], indexes that quantify performance in relation to publications and citations provide a convenient currency that increasingly is used to approximate the overall impact of a researcher [3]. Perhaps not surprisingly given the demand and ease with which information on publication and citation records is obtained, the number and variety of these “bibliometric” indexes has exploded in the last decade (reviewed by [3–6]). A necessary condition for effective use of such metrics is an ability to place values into an appropriate context [7]. The fact that a faculty member averages 35 citations per year is meaningful only to the extent that her performance can be compared to a reference group. Numerous studies have established performance benchmarks for particular disciplines to enable comparison to peers [7–13] or to highly regarded scientists [14–16]. Here, we provide a model-based process to benchmark performance of faculty in fisheries and wildlife disciplines.

Although the notion of benchmarking is intuitive, it can be difficult to implement. Within a discipline, two faculty members may differ in a bibliometric score for reasons that have nothing to do with innate differences in their ability or the quality of their work. For instance, the number of years over which a scientist has been active in a chosen profession is related positively or in an asymptotic fashion to indexes that rely on accumulated counts of publications or citations [17,18] and negatively to indexes expressed as rates that are influenced by temporal trends in publication and citation practices [19]. In addition, few faculty members devote all of their time to research; instead, many have responsibilities associated with teaching, extension and outreach, or service. Not surprisingly, those with more non-research related responsibilities tend to produce lower bibliometric scores than faculty with greater research appointments [20]. Sex differences in bibliometric scores also are common within a discipline; in several disciplines males tend to generate higher performance scores than females [10,15,21]. Even within a discipline, sub-disciplines can differ dramatically in publication and citation practices [22]. Despite the importance of these covariates on bibliometric scores, most benchmarking studies have failed to incorporate their effects or focused on a single covariate, typically professional age [9], but see [15].

One approach to account for such covariates is to control for them statistically: “The residuals of a regression of productivity on age and other control variables might provide a promising avenue for a tailor-made individual adjustment for every individual in the sample” [18]. We apply a standardized residual approach to regression models of bibliometric performance by fisheries and wildlife faculty that incorporate potentially influential covariates. We also consider the feasibility of comparing performance across various scientific sub-disciplines within fisheries and wildlife.

## Materials and Methods

### Benchmarking against peer performance

In a separate study [20], we identified ‘best’ models to predict publication and citation metrics for 437 faculty at 33 research-extensive universities in the United States that were members of the National Association of University Fisheries and Wildlife Programs (NAUFWP). For each of 437 faculty members we collected bibliometric information from Web of Sciences searches and obtained information on faculty appointments by contacting institutional administrators. We determined Ph.D. date for faculty from department webpages and by searching for electronic dissertations in ProQuest database. For faculty with common last names, we refined searches as described in [20] and compared results to personal web pages and publication lists. Sub-disciplines of research were assigned after examination of faculty webpages and selected publications. Finally, we developed regression models for Hirsch’s h-index and m quotient [14], the h_b_-index [23], number of publications, and annual citation rate [20]. An individual’s h-index is defined as the number of publications with at least *h* citations each [14]. Hirsch argued that two individuals with the same h-index were similar in terms of overall scientific impact, even if they differed in excess citations (or excess publications) [14]. Brown disagreed and defined the h_b_ index as $h_{b}\;=\;h+\sqrt{e}$, where *e* is the sum of all citations in excess of *h*^2^ for those articles that contribute to *h* [23,24]. Hirsch [14] further defined the m quotient as the annual rate of increase in *h* over the years of scientific activity for an individual. For m quotient and annual citation rate, we equated years of scientific activity to years since conferral of the Ph.D. [20]. For the population of 437 faculty members, significant variation in some or all of these performance metrics was explained by time allocated to research (i.e., research appointment percentage), elapsed years since Ph.D. conferral (i.e., academic age), a quadratic term associated with academic age, sex, the sub-disciplines in which research was conducted (8 sub-disciplinary categories were incorporated into models as indicator variables to which a faculty member could be assigned—disease, genetics, social sciences, management, ecology, quantitative, conservation and aquatic sciences), and how early the first publication appeared in one’s academic career [20].

The generalized linear models developed previously [20] form the basis for our benchmarking approach. Herein, we develop similar models for three additional performance metrics. Total citations were included as a cumulative measure of the impact of an individual’s scholarship [3]. The median number of citations for publications in the h-core, i.e. the m index, was included because it has been recommended as a measure of impact that complements h [3,5]. Finally, the r index, defined as the square root of the sum of citations for papers with h or more citations [25], was computed to measure citation intensity in the h core.

Details on model development are provided elsewhere [20]. Briefly, we constructed a set of nested candidate models, with an intercept-only model followed by sequential incorporation of academic age (and a centered quadratic term if deemed appropriate after inspection of residuals), percent of appointment allocated to research, sex (0 = female, 1 = male), and sub-discipline. Unlike some previous analyses [20], we did not include the difference between the years of Ph.D. conferral and first publication as a covariate, because doing so to benchmark using standardized residuals would unjustly penalize individuals for publishing at an early career stage. Negative binomial models were fitted, after rounding to the nearest integer, for h, h_b_, m, and r indexes, number of publications, number of citations, and annual citation rate, computed as the quotient of citation count and elapsed years since attainment of Ph.D. Models were fitted with function glm.nb from the MASS library [26] in R 3.0.2 [27], as considerable over-dispersion was evident in models fitted to a Poisson distribution. Gaussian regression models were fitted for the m quotient, which we defined as the quotient of h-index and years since Ph.D. conferral [5], after transformation of academic age to ln(age + 0.5) to reduce heteroscedasticity. We set academic age to zero for one individual who obtained the Ph.D. in 2015. Nested models were compared using likelihood-ratio tests to determine which covariates to retain, and a final model was fitted to each of the response variables for benchmarking purposes.

To benchmark against peer performance, the final models were used to compute standardized deviance residuals for each of the 437 faculty members [28], where deviance is the difference in log likelihoods between a proposed model and a saturated model with one parameter fitted for every observation in the dataset. Standardized deviance residuals weight each observation’s deviance by the influence of the observation on the model [29]. The standardized residuals form the basis for comparisons among peers, as they represent deviations in performance after accounting for variation due to academic age, sex, and other factors. The sign of the residual indicates direction of deviation from expectation. Positive and negative residuals suggest better and worse performance relative to model prediction, respectively. More specifically, the standardized residual for an individual faculty member can be compared to a histogram or distribution of standardized residuals for all faculty members to assess the percentile of the distribution with which she is associated. For each model we ranked faculty in descending order of standardized residuals to generate percentile rankings. For a large enough sample size in regressions, internally standardized residuals approximate a standard normal distribution [28,29]. Thus, we also computed percentile rankings for faculty based on a standard normal curve.

When considered as an evaluation tool, use of multiple indicators is recommended to provide a more accurate depiction of performance [3,30]. Thus, we also used the standardized residuals to compute benchmarks for individual faculty by combining results across models. First, we used the percentiles of standardized residuals for the 8 metrics to compute the mean, range, and 95% confidence interval of percentile ranks for each faculty member. Equal weighting of individual metrics to compute a mean percentile is most appropriate when metrics are uncorrelated, but some of our measures exhibited strong correlations (S1 Table). Therefore, in a second approach, we reduced the dimensionality of the standardized residuals via a factor analysis with varimax rotation. The resulting factor scores were displayed in two dimensions, with convex hulls for the innermost 100, 95, 90, 75, and 50 percent of factor scores superimposed on bivariate plots. We computed a joint percentile based on a mean factor score for each faculty member that was weighted by the relative proportion of variation explained by each factor. To facilitate the use of our benchmarking approach by other individuals, including faculty not in the population for which models were developed, we provide R code to perform the modeling and computations described above, given a set of values for response variables and covariates (S1 Code).

To illustrate the use of these models as a benchmark for peers, we consider a hypothetical male associate professor of fisheries and wildlife 15 years post-Ph.D. that conducts ecologically based research on fish diseases (Table 1). We also assume that the individual develops quantitative tools associated with analyses used in disease ecology. The faculty member would thus be categorized as active in the sub-disciplines of ecology, disease, and quantitative sciences. Further, suppose that the faculty member has 25% effort allocated to research. In terms of publication and citation metrics, suppose the citation record for the faculty member, for 30 publications ranked in descending order of number of citations, is (50, 40, 30, 20, 18, 16, 15, 14, 12, 10, 9, 8, 6, 5, 5, 4, 3, 1, 1, 1, 1, 1, 0, 0, 0, 0, 0, 0, 0, 0) (Table 1). Ten publications have 10 or more citations, thus the h-index is 10. The minimum number of citations needed for an h-index of 10 is 10^2^ = 100, so the number of excess citations in the h core is e = 225–100 = 125, and h_b_ = 10 + 125^1/2^ = 21. The m quotient is the ratio of h-index and academic age, so m quotient = 10/15 = 0.67. The annual citation rate is 270/15 = 18. The median number of citations for papers appearing in the h-core, i.e., the m index, is (18+16)/2 = 17. The r index is 225^1/2^ = 15.

**Table 1:** Standardized Deviance Residuals and Benchmarking Percentiles for Hypothetical Faculty Member in Fisheries and Wildlife.

Metric	Value	Residual	Benchmarking Percentiles^a^
**h-index** [14]	10	-1.05	16.0
**h**_**b**_**-index** [23]	21	-1.40	9.6
**m quotient** [14]	0.67	-0.96	9.1
**Publications**	30	-1.02	17.8
**Citations**	270	-1.36	12.8
**Citations/year**	18	-1.48	11.0
**m index** [3,5]	17	-1.46	8.7
**r index** [26]	15	-1.34	11.2
**Mean/Percentile**		-1.26	12.0/8.4

Residuals and percentiles computed from regression models for a hypothetical faculty member with the bibliometric values listed above and the following characteristics: male, 25% research appointment, and a sub-disciplinary research focus in ecology, disease, and quantitative methods.

^a^Benchmarking percentiles represent the percentage of all faculty in the database (n = 438) with residuals as small or smaller than the residual of the hypothetical faculty. The value of 12.0% averaged across the 8 bibliometrics is as large as 8.4% of averages for other faculty.

### Benchmarking institutions

Aggregation of individual-level performance metrics can be used to make comparisons across institutions (e.g., [18,31]). For each performance metric we ranked universities with at least five faculty in fisheries and wildlife according to their mean standardized residuals. For each university we then depicted with a boxplot the median, interquartile width, and range of the mean ranks of residuals across the 8 performance metrics. We assessed the importance of university affiliation as a covariate in regression models using likelihood ratio tests.

### Benchmarking across disciplines

Faculty in fisheries and wildlife are heterogeneous with respect to training and research interests, with disciplinary ties that range from genetics to ecology to social sciences. Publication and citation practices differ substantially across disciplines [30]. Hence, comparison of faculty from different disciplines requires normalization that accounts for variation in citation and publication practices. We believe that our regression approach provides an improvement over prior attempts at normalization when considering peers within a discipline (fisheries and wildlife in this case). As one comparison, we extended the ‘law of constant ratios’ [32] to disciplines with which faculty and fisheries and wildlife are aligned.

Podlubny examined the number of citations produced in each of 6 years spanning the period 1992–2001 for 9 different disciplines [32]. He found that even though the number of citations in the disciplines increased over time, the ratio of citations for any two disciplines remained stable over time. Consequently, he suggested a simple normalization, i.e., the ratio of total citations in a discipline to total citations in mathematics (chosen as a reference), to enable comparisons of scientists from different disciplines. Subsequent study has shown that normalization to a mean number of citations per article published per year works as well or better than more complicated scaling methods [33]. Thus, we collected number of citations per article per year for the fisheries and wildlife sub-disciplines examined previously [20] and as classified in the Web of Science Journal Citation Reports (JCR). Specifically, we mapped 18 JCR categories to 7 of the 8 sub-disciplines used in our benchmarking models (Table 2); no JCR categories characterized our management sub-discipline. Following convention [16,32], we used the field of mathematics as a reference and computed the ratios of number of citations per article per year for each mapped set of JCR categories relative to mathematics. We computed these ratios for every year from 2005 to 2014 and used linear regression of year (centered) to determine whether the ratio remained constant across years, with interaction terms to test whether sub-disciplines differed in temporal trends of citations/article (relative to mathematics). As per the Purdue University Senior Protocol Analyst, Human Research Protection Program, this study does not qualify as human subjects research as defined by the U.S. Department for Health and Human Services and hence was not subjected to IRB review.

**Table 2:** Classification Used to Map Journal Citation Report^®^ Categories onto Sub-disciplines in Fisheries and Wildlife.

Sub-disciplines	Journal Citation Report Categories
**Disease**	Infectious diseases, Parasitology, Environmental sciences, Nutrition & dietetics, Anatomy & morphology
**Genetics**	Genetics & heredity, Evolutionary biology, Biochemistry & molecular biology
**Social sciences**	Sociology, Social sciences interdisciplinary, Agricultural economics & policy
**Ecology**	Ecology
**Quantitative**	Statistics & probability, Remote sensing
**Conservation**	Biodiversity conservation
**Aquatic sciences**	Limnology, Fisheries, Marine & freshwater biology

## Results

### Benchmarking against peer performance

All final models included academic age, percent of appointment allocated to research, and sub-discipline (Table 3). Number of publications was the only response variable for which sex was significant (Table 3). Models reduced deviance by 25.2–52.4% relative to intercept-only models (Table 3).

**Table 3:** Fitted Models for Eight Bibliometrics of Scholarly Performance by Fisheries and Wildlife Faculty.

	Bibliometric Response Variable Coefficients^a^ (Standard Error)							
Covariate	h-index^b^	h_b_-index	m quotient	Publications	Citations	Citations/year	m index	r index
**Intercept**	**1.48** (.09)	**2.35** (.09)	**2.08** (.18)	**2.40** (.13)	**4.23** (.17)	**2.60** (.16)	**2.27** (.10)	**1.92** (.09)
**Academic age**	**0.04** (.002)	**0.04** (.002)	**-0.52** (.04)	**0.05** (.003)	**0.09** (.004)	**0.02** (.004)	**0.04** (.002)	**0.04** (.002)
**Age**^**2**^	**-0.001** (.0002)	**-0.001** (.0002)		*-0*.*001* (.0002)	**-0.002** (.0004)		**-0.001** (.0002)	**-0.001** (.0001)
**Research**	**0.01** (.001)	**0.01** (.001)	**0.005** (.001)	**0.008** (.001)	**0.01** (.002)	**0.01** (.002)	**0.004** (.001)	**0.01** (.001)
**Sex**				*0*.*21* (.08)				
**Disease**	**0.21** (.06)	**0.22** (.06)	*0*.*18* (.07)	**0.29** (.08)	**0.43** (.12)	**0.42** (.12)	*0*.*18* (.07)	**0.21** (.06)
**Genetics**	**0.27** (.07)	**0.30** (.08)	**0.30** (.09)	*0*.*26* (.10)	**0.63** (.14)	**0.61** (.14)	**0.34** (.08)	**0.32** (.07)
**Social**	**-0.25** (.08)	**-0.26** (.08)	*-0*.*23* (.09)	*-0*.*21* (.10)	**-0.48** (.15)	**-0.45** (.14)	**-0.29** (.08)	*-0*.*25* (.08)
**Management**	**-0.22** (.04)	**-0.20** (.05)	**-0.20** (.06)	*-0*.*13* (.06)	**-0.39** (.09)	**-0.38** (.09)	**-0.21** (.05)	**-0.23** (.05)
**Ecology**	**0.15** (.05)	**0.18** (.05)	*0*.*12* (.06)	0.11 (.07)	**0.33** (.10)	**0.34** (.09)	**0.23** (.05)	**0.21** (.05)
**Quantitative**	*0*.*18* (.06)	**0.24** (.06)	*0*.*16* (.07)	0.11 (.08)	**0.50** (.12)	**0.50** (.11)	**0.30** (.06)	**0.28** (.06)
**Conservation**	*0*.*10* (.05)	*0*.*13* (.05)	0.07 (.06)	0.07 (.07)	*0*.*27* (.10)	*0*.*27* (.09)	**0.18** (.05)	*0*.*16* (.05)
**Aquatics**	0.16 (.09)	018 (.10)	0.12 (.12)	0.08 (.14)	*0*.*43* (.20)	*0*.*39* (.19)	*0*.*29* (.11)	*0*.*22* (.10)
**θ**	9.17 (1.04)	5.40 (0.44)	na	2.84 (0.20)	1.28 (0.08)	1.47 (0.10)	5.05 (0.41)	6.21 (0.54)
**Improvement in Deviance**^c^	52.4%	48.4%	43.4%	44.5%	47.0%	25.2%	42.4%	49.2%

Academic age = years since conferral of Ph.D.; ln(academic age + 0.5) was used to model m quotient. Age^2^ = square of age, after centering; Research = percentage of appointment allocated to research; Sex = 0 if female, 1 if male; θ is the over-dispersion parameter for negative binomial models (all except m quotient, which was fitted via linear regression). Disease, …, Aquatics are binary variables for the 8 sub-disciplines.

^a^Boldface signifies p < 0.001; italics signifies p < 0.05.

^b^The predicted h-index for a faculty member of average academic age (= 18.6 years) specializing in genetics and with a 52% research appointment is computed as exp(1.48 + .04*18.6 + .01*52 + .27*1) = 20. In this example, age^2^ is 0 since the variable age^2^ is centered on the mean and the faculty member is assumed to be of average academic age. No exponentiation is applied to the linear regression for m quotient. Additional examples are given in [20]. However, note that the coefficients and models in [20] include publication precocity (or year of researcher’s first publication relative to PhD attainment) as a variable.

^c^Relative to intercept-only model.

After appending vectors of covariate values and bibliometric values for the hypothetical faculty member to the corresponding vectors for the 437 faculty members in our population (S1 Data), we refit the final models to obtain coefficients that incorporate data for the hypothetical faculty member and enable computation of standardized residuals (S1 Code, S2 Data). Of the metrics, the m quotient was least correlated with other variables (S1 Table) and hence may merit special attention. In our example, the hypothetical individual exhibited a standardized residual of -0.96 for m quotient and -1.05 for Hirsch’s h-index (Table 1), corresponding to percentile rankings of 9.1% and 16.0%, respectively, for these two complementary variables [14]. When all 8 bibliometrics were considered, the hypothetical faculty member exhibited an average standardized residual of -1.26. His average percentile rank of 12.0 across the 8 metrics is greater than only 8.4% of peers (Table 1). A similar value, 10.4%, is obtained for the percentile of a standard normal variable with a value of -1.26. Across all faculty, percentiles obtained empirically and via the normal approximation were highly correlated (r = 0.989, p << 0.0001).

**Fig 1:**
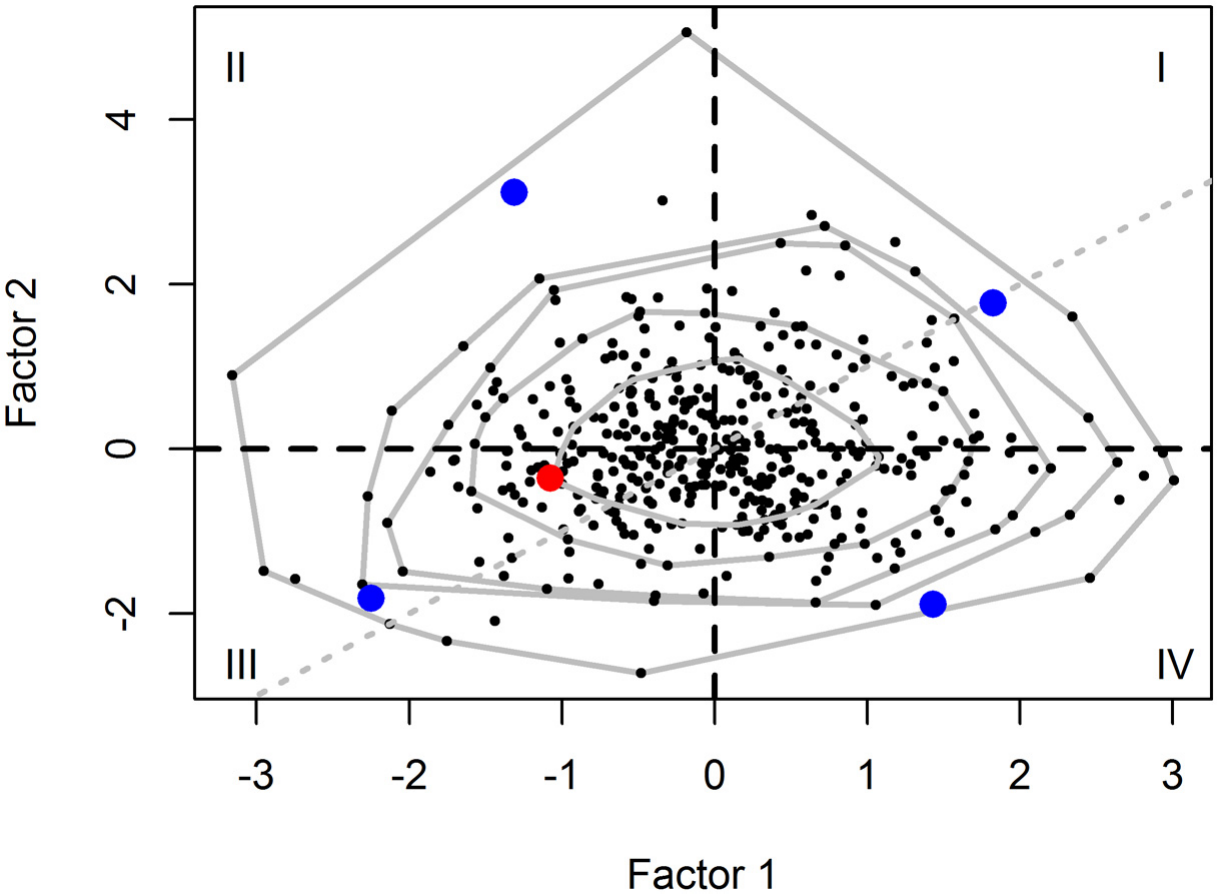
Benchmarking of faculty performance with factor analysis on residuals from models for 8 bibliometric variables. Factor 1 is correlated most highly with performance metrics that emphasize citation counts, whereas Factor 2 is most correlated with publication count. Convex hulls are superimposed for the innermost 100, 95, 90, 75, and 50 percent of factor scores to facilitate comparison. The dashed 45-degree line represents performance that is equally good (Quadrant I) or poor (Quadrant III) for both factors. The hypothetical faculty member (Table 1) is depicted with a red circle in Quadrant III. Representative faculty between the 95 and 100 percent hulls are highlighted in each quadrant with blue circles.

For individual faculty, standardized deviance residuals from the models of the 8 bibliometrics exhibited considerable correlation (S1 Table). A factor analysis produced two factors that explained 53.8% and 34.2% of the variation, respectively. Factor 1 was correlated most highly with the performance metrics that emphasize citation counts, whereas Factor 2 was most correlated with publication count (Table 4). The h-index and m quotient exhibited correlations that were similar between the factors (Table 4). Our hypothetical faculty member exhibited scores that indicated below-average performance for both factors relative to peers; i.e., he fell in quadrant III of the bivariate scatter plot (Fig 1). Indeed, only 12.1% of peers scored at least as low on factor 1 (score ≤ -1.07), whereas 35.8% scored at least as low on factor 2 (score ≤ -0.36). To consider the factors jointly, we computed a mean score weighted by the relative variation explained by each factor. The first factor accounted for .538/(.538 + .342) = .611 of explained variation, whereas the second factor accounted for the remaining .389. Thus, the weighted mean for the hypothetical faculty member is -1.07(.611) + -0.36(.389) = -0.80, yielding a ranking of 48, i.e., 11.0% of peers produced a joint weighted score at least as low. For the faculty highlighted in blue in each quadrant (Fig 1), the percentiles computed from the weighted linear combination of factor scores are 99.5 (quadrant I), 73.0 (II), 0.9 (III), and 61.6 (IV). Rankings from the two aggregative approaches, i.e., factor analysis and the unweighted means of percentiles, were highly correlated (r = 0.985, p << 0.0001).

**Table 4:** Factor Analysis on Standardized Deviance Residuals from Regression Models of Bibliometric Performance by Fisheries and Wildlife Faculty.

	Factor Loadings	
Bibliometric	Factor 1	Factor 2
**h-index**	0.703	0.648
**h**_**b**_**-index**	0.779	0.598
**m quotient**	0.486	0.430
**Publications**	0.339	0.895
**Citations**	0.801	0.593
**Citations per year**	0.794	0.577
**m index**	0.893	0.284
**r index**	0.884	0.457
**Proportion of variation explained**	0.538	0.342

Residuals for each bibliometric variable were computed from models in Table 3.

### Benchmarking institutions

**Fig 2:**
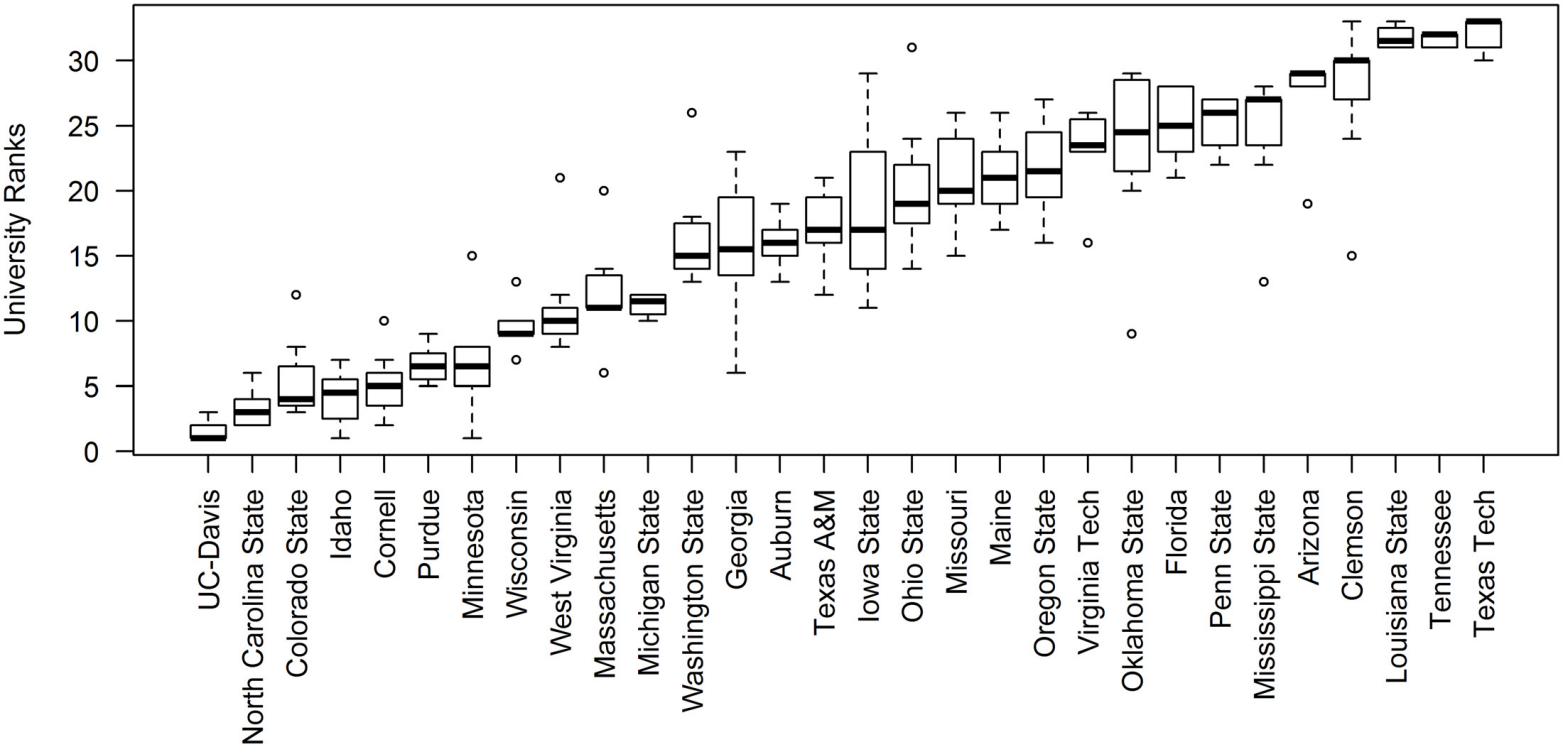
Performance metrics of fisheries and wildlife faculty vary with university affiliation. The box plot depicts median, interquartile width, and range of the mean ranks (1 = best) of standardized deviance residuals across 8 performance metrics for 30 universities as computed from models fitted to data for 437 faculty. Three universities (Connecticut, Kentucky, Nebraska) are not shown because they had fewer than five faculty members in fisheries and wildlife.

Likelihood-ratio tests indicated that university affiliation explained significant amounts of variation among institutions for all performance metrics except m quotient. When average standardized residuals were considered as ranks for the 8 performance metrics, University of California-Davis exhibited the best median ranking across the 8 metrics, followed by North Carolina State University and Colorado State University (Fig 2). Some programs (e.g., Cornell University, Colorado State University, University of Minnesota) with strong median ranks exhibited substantial variation in rankings across the 8 metrics, whereas other highly ranked programs (e.g., University of California-Davis, North Carolina State, Purdue University) were notable for consistency in rankings and hence low variation across the metrics (Fig 2).

### Benchmarking across disciplines

**Fig 3:**
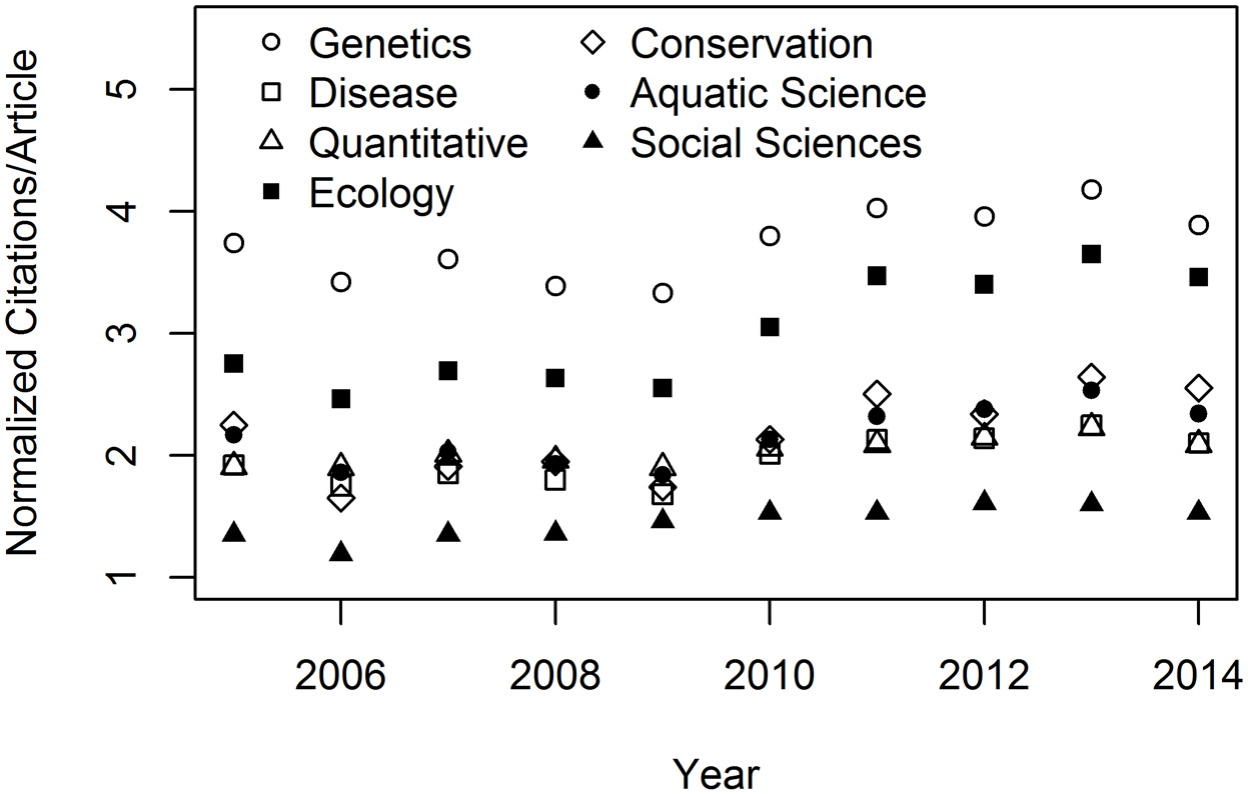
Citation practices in fisheries and wildlife sub-disciplines do not obey the law of constant ratios. Eighteen categories from Web of Science Journal Citation Reports^®^ were clustered into the 7 sub-disciplinary categories (Table 2) to derive annual averages for citations per article, normalized to mathematics. Ratios increased over time, with comparable rates except for ecology.

When the mapped set of JCR categories was considered (Table 2), annual values for citations per article from 2005–2014 were 1.19 to 4.18 times greater than the citations per article in mathematics. The normalization ratios differed significantly between genetics and all other sub-disciplines (all P << 0.001), with highest values for genetics and lowest values for social sciences (Fig 3). Ten-year mean ± 1 SD values for normalization ratios were 3.73 ± 0.29 for genetics, 3.01 ± 0.45 for ecology, 2.17 ± 0.35 for conservation, 2.15 ± 0.24 for aquatic sciences, 2.03 ± 0.11 for quantitative, 1.96 ± 0.19 for health sciences, and 1.45 ± 0.13 for social sciences. A significant increase in normalization ratios occurred over time (t = 3.32, df = 56, p = 0.002), indicating that citations per article for the disciplines in question increased relative to mathematics over the 10-year period (Fig 3). Decadal sets of annual normalization ratios were highly correlated for all pairs of disciplines (mean r = 0.90, range: 0.75–0.98). Moreover, disciplines did not differ in the rate at which their normalization ratios changed relative to genetics (all p > 0.20), except for ecology (t = 2.28, df = 56, p = 0.03). Ecology experienced a more rapid increase in its normalization ratio than genetics, driven primarily by increases from 2010–2014 (Fig 3).

## Discussion

### Benchmarking basics

Our approach to benchmarking differs from previous studies because it explicitly attempts to account for variation in publication and citation metrics due to academic age, sex, research appointment, and sub-disciplinary focus. Collectively, these covariates reduced deviance by roughly ¼ to ½, although sex was only important when predicting number of publications. Such levels of explanatory power may seem modest, but we contend that incorporation of these covariates into benchmarking efforts is a marked improvement over prior methods because doing so enables comparison of individuals with differing academic age or research appointment and focus. Career duration has long been recognized as a factor that influences performance metrics, especially those that increase monotonically over time [14,19,34,35]. Prior benchmarking studies occasionally have accounted for age effects by conducting cohort analyses, but this approach is inferior to one that uses covariates to allow for “tailor-made” adjustments for every individual [18]. For soil scientists, a simple linear regression of h-index against academic age was provided, but other covariates were not considered nor were residuals used for comparisons of individuals [9]. A regression of h-index against academic age and sex has also been published [15], but for a sample limited to members of editorial boards of seven evolution and ecology journals. Likewise, sub-disciplinary effects have been noted in other fields [22,36,37], but attempts to account for variation due to sub-disciplinary focus thus far have relied on reference groups such as Nobel laureates [22] or named professors [20]. We are unaware of attempts to incorporate a faculty member’s research appointment, perhaps because this information is not provided on bibliometric search engines.

Standardized residuals offer a convenient statistic with which to compare discrepancies between observed and expected performance. At the level of individual faculty, the simplest approach is to compute standardized residuals and associated percentiles for each performance metric. We presented models for 8 metrics because each has its own strengths and supporters. Our hypothetical faculty member ranked ahead of only 8.7% of the 437 “peers” with whom he was compared using the m index, but he ranked ahead of 17.8% of them when number of publications was considered (Table 1). If general trends are more important than consideration of individual metrics, a mean percentile can be computed across all 8 metrics for each faculty member, or for some subset of metrics of interest. Such an approach provides an overall benchmark of performance as well as an indication of the degree to which the performance varied among the metrics under consideration. For the hypothetical faculty member, the overall mean performance ranked ahead of 8.4% of peers and was fairly consistent across the 8 metrics (95% CI: 6.2–11.6%).

Factor analysis is a more sophisticated approach with the same goal as above, namely, to express performance meaningfully in a reduced number of dimensions. Factor scores have been used to demonstrate the utility of performance metrics in predicting scores derived independently via peer assessment for applicants to a long-term fellowship program in molecular biology [38,39]. We interpret the factors extracted from our data as differentiating between faculty who publish more articles and hence are more productive (Factor-2) and faculty who have higher citations or impact independent of productivity (Factor-1). Our 2 factors explained 88% of the variation in the standardized residuals for the 8 metrics (Table 4). The hypothetical faculty member fell within the convex hull containing 75% of all faculty members, but his performance was below average for both factors (Fig 1). Indeed, joint consideration of the two factors reveals that only 11.0% of faculty exhibited a weighted mean score as low or lower than the hypothetical faculty member. In general, the quadrant in which a faculty member resides conveys information on the joint distribution of the factor scores. Quadrant I is occupied by faculty who exceed the average in productivity and citations, whereas Quadrant III is populated by faculty who fall below the average in both of these dimensions. Quadrant II contains faculty members who produce more publications than average but fewer citations, i.e., they exhibit performance that emphasizes quantity over impact on peers. Although they publish as much as their otherwise comparable peers in Quadrant I, each of their articles is cited less. Finally, Quadrant IV contains faculty who produce fewer-than-expected publications but greater-than-expected citation metrics. Faculty members in Quadrant IV seem to emphasize impact of publications, as measured by citation metrics, over quantity. Although they produce fewer publications than their otherwise comparable counterparts in Quadrant I, each of their publications receives more attention from peers.

Faculty members exhibit considerable variation in performance after accounting for variation explained by age, research sub-discipline(s), percent research appointment, etc. Far less variation was noted among institutions, although some differences were evident (Fig 2). Students and prospective faculty should exercise caution when examining these metric-based rankings of institutions. They represent a snapshot of performance that will change as faculty retire and are hired. Moreover, they are silent on many of the other factors that are important to quality educational and career goals including social climate, administrative support, curricular offerings, and depth of expertise, to name a few [40]. Nonetheless, they do suggest that expectations for performance can be institutionalized to some degree, perhaps due to differences in infrastructure and research support, reward systems, or the standards used for hiring, promotion and tenure processes.

Normalized citations per year were not constant over time for the sub-disciplines affiliated with fisheries and wildlife (Fig 3), in contrast to previous work [32]. However, the rate of change in citations per article relative to mathematics did not differ across sub-disciplines, except for ecology. Thus, a “quick-and-dirty” comparison between disciplines other than ecology is possible for those who eschew model-based benchmarking. To illustrate, suppose that faculty member X in genetics has 1000 citations and 100 publications (10 cites/article), whereas faculty member Y in social sciences has 500 citations and 100 publications (5 cites/article). The raw metric for citations per article indicates that faculty X produces cites/article at twice the level of faculty Y. But the normalization ratios, 3.73 for genetics and 1.45 for social science, suggest that faculty Y has had greater impact than faculty X, after taking into account differences in citation and publication practices between disciplines. Specifically, when considered in reference to mathematics, faculty X produces 10/3.73 = 2.68 cites/article, whereas faculty Y produces 5/1.45 = 3.45 cites/article. The application of this approach should be limited to broad comparisons that inform discussion among academic units, because, as we have shown, other factors (sex, research appointment, academic age) can exert effects independent of discipline.

### Benchmarking caveats

We believe that results from our benchmarking study are useful as indicators of past performance based on citations or publications after accounting for variation due to academic age, sex, research appointment, and sub-disciplinary focus. However, they represent indices of performance at a particular point and should not be used to forecast future success in academia. Cumulative measures of achievement, like the h-index and its variants, tend to be poor predictors of future success due to temporal fluctuations in productivity [41]. Importantly, assessment of predictive power of regressions for such indexes is invariably inflated by their inherent autocorrelation [42]. Higher levels of variation in m-quotient values for faculty early in their academic careers also limits its utility for prediction with this group [20]. If the intent is to predict future success, a focus on rates of past achievement may be more appropriate [41,43]. Moreover, the definition of success may influence the predictive utility of various metrics. For >25,000 scientists in PubMed, the probability of becoming a principal investigator was modeled with relatively high accuracy (area under the logistic regression curve = 0.83) as a function of performance metrics such as number of first-authored publications, number of publications, and h-index [44]. When success was defined as the number of publications produced over the decade following conferral of a Ph.D., the number of prior publications and an earlier date of first publication relative to the Ph.D. were positive predictors of success for a sample of academics in biological and environmental sciences, but the best models only explained about 15% of the deviance [21].

Estimates from our regression models are of course dependent on the accuracy of bibliometric and covariate data used in analyses. We relied on data from the Web of Science to compile the data from which response metrics were computed. We chose Web of Science because it is used routinely. However, it is important to note that rankings produced by data from Scopus or Google Scholar may differ from Web of Science due to differences in the database coverage [45,46]. For any given database, blind reliance on bibliometrics computed automatically by search engines can lead to inaccuracies caused by errors of omission or commission [47]. To minimize this problem, we followed the recommendation [47] to conduct partially manual searches [20]. Still, we suspect that accuracy declined for more senior faculty in our sample, presumably corresponding to poorer coverage of old publications by Web of Science. We attempted to compensate for this perceived bias by conducting more individualized searches for these faculty, but errors may still have occurred.

Inaccuracies also could affect data collected for covariates. For instance, we relied on administrators from the target universities to provide information on research appointments of faculty. At least three factors could increase noise in these data. First, accounting practices associated with research appointments may differ among institutions. Second, research appointments may change over time; in our study we used the most recent appointment unless information provided in correspondence with administrators indicated that doing so was inappropriate due to a faculty member’s much longer period of service in a different research appointment. Finally, faculty appointments may not accurately reflect how a faculty member allocates time to research versus other activities. Given these caveats, it is noteworthy that we found strong, consistent effects of research appointment for all metrics considered.

Our assignment of faculty according to sub-disciplines was subjective and based on assessment of websites and publications. Hence, it certainly is possible that faculty were misclassified in some instances. Moreover, our sub-disciplinary categories may themselves represent heterogeneous mixtures of research focus. For example, the quantitative sub-discipline included research on quantitative methods and on geospatial science, even though it could be argued that these warrant separate sub-disciplinary designations. Future attempts to incorporate sub-disciplinary effects could benefit from more objective classification, perhaps using visualization of key words [37]. Still, the similarity in citation rates for JCR categories that were mapped onto individual sub-disciplines (S2 Table) provides some support for our classification. Individual faculty members are likely to work in multiple sub-disciplines [20], with associated effects on benchmarking metrics; indeed, a unimodal relationship has been shown between research breadth and h-index [48]. In situations where benchmarking is desired for faculty who are difficult to categorize using our sub-disciplinary groupings, regression models can easily be re-fitted without sub-disciplinary covariates.

We believe that our method improves on earlier efforts at benchmarking because it addresses several of the issues raised in the Leiden Manifesto [30]. For instance, those who rely on quantitative indicators of productivity and impact should choose methods that account for variation in citation and publication practices across fields of inquiry, adopt multiple indicators to offer a more well-rounded description of performance, and ensure transparency in data and analyses [30]. Nonetheless, we have devoted considerable discussion to caveats of our benchmarking method because, to be blunt, quantitatively based rankings of performance are prone to abuse. We strongly agree with other researchers [30] that quantitative benchmarks should supplement, not supplant, expert assessment of productivity and impact by an individual’s peers. Similar to the caution provided by Engqvist and Frommen [49], it would be inappropriate to base important decisions (e.g., hiring, promotion, allocation of grant funds) on small differences in rankings from our benchmarking models. Even if our models could perfectly account for variation in publication and citation metrics, they should not replace critical evaluation by peers. A faculty member’s reputation often is built on more than publications and citations; for instance, broader impacts that affect individual behavior or social policy may not necessarily be related to bibliometrics [3]. And of course our models, as with all models, are imperfect simplifications of reality. While models can provide important insights into performance, they are silent on numerous sources of variation in performance that may be recognized only by knowledge gained from careful inspection of an individual’s record. Such sources of variation for an individual researcher may include details on the specific research questions asked and approaches used, philosophies on student mentoring and co-authorship, levels of commitment to professional service, or institutional inequities in research support. Our benchmarking methods can provide crucial information, but they should be viewed as one set of tools in a comprehensive evaluation system [30].

## Supporting Information

S1 Code Manual and R (3.0.2) code to perform comparisons and analyses with additional faculty members. (PDF)

S1 Data Database of 437 faculty used for model-based benchmarking of fisheries and wildlife. (CSV)

S2 Data Sample database of “new” individuals for inclusion in analysis after re-fitting of models with S1 Code. (CSV)

S1 Table Correlations of standardized deviance residuals from 8 models for faculty in fisheries and wildlife. (DOCX)

S2 Table Average annual normalized citations per article for JCR categories and associated fisheries and wildlife sub-disciplines as grouped in Table 2. Normalization was accomplished by dividing values by the corresponding citations per article for mathematics.
(DOCX)
