# Supplementary material for: Performance Benchmarks for Scholarly Metrics Associated with Fisheries and Wildlife Faculty
Source: PLoS One. 2016 May 6;11(5):e0155097. doi: 10.1371/journal.pone.0155097 (PMC4859475; doi:10.1371/journal.pone.0155097)
Supplement: S1 Code — (PDF) [file pone.0155097.s001.pdf]

NAUFWP Faculty Benchmarking Analysis

Kenneth F. Kellner and Robert K. Swihart

Introduction

This document provides instructions for calculation of standardized deviance residuals for the original data presented in the PLoS One paper by Swihart et al., with the option of adding new individual(s) to benchmark.

Data Input

First, set the working directory to the location of the full faculty database ('*S1_Data.csv*') and make sure the required R packages are installed and loaded. If any of the packages are missing, they will be downloaded and installed automatically.

```
setwd(choose.dir())
```

```
if(!'MASS'%in%installed.packages()[,'Package']){install.packages('MASS')}
if(!'aplpack'%in%installed.packages()[,'Package']){install.packages('aplpack')}
require(MASS); require(aplpack)
```

Next, read in the database, and inspect the result in R:

```
alldata <- read.csv('S1_Data.csv',header=TRUE)
```

The database contains a single row for each scientist. Columns 1-11 are the independent variables:

```
head(alldata[,1:11])
```

	age.phd	Male	Re	health	genetics	social	applied	ecology	quant	conservation	aquaticsci
1	41	1	10	0	0	1	0	0	0	0	0
2	12	1	50	1	0	0	0	1	0	1	0
3	4	1	65	1	0	0	0	1	0	0	0
4	4	1	60	0	0	0	0	1	1	0	0
5	19	0	50	1	0	0	0	0	0	0	0
6	6	1	70	0	0	0	1	1	0	1	0

1. *age.phd*: Academic age, i.e., the number of years since conferral of the Ph.D.
2. *Male*: Sex (0 = female, 1 = male)
3. *Re*: Percent of appointment allocated to research (0-100)
4. *health*: Disease-related subdiscipline? 0 if no, 1 if yes
5. *genetics*: Genetics subdiscipline? 0 if no, 1 if yes
6. *social*: Social sciences? 0 if no, 1 if yes
7. *applied*: Management-related subdiscipline? 0 if no, 1 if yes
8. *ecology*: Ecology subdiscipline? 0 if no, 1 if yes
9. *quant*: Quantitative subdiscipline? 0 if no, 1 if yes

10. *conservation*: Conservation-related subdiscipline? 0 if no, 1 if yes
11. *aquaticsci*: Aquatic sciences subdiscipline? 0 if no, 1 if yes

Columns 12-19 are response variables:

```
head(alldata[,12:19])
```

	nPubs	h	mqPhD	hb	citesperyrPhD	nCites	mindex	rindex
1	39	8	0.195	18.392	4.195	172	11.0	10.536
2	34	9	0.750	20.091	17.000	204	12.0	12.000
3	27	8	2.000	22.526	68.750	275	27.5	15.811
4	17	7	1.750	16.695	35.750	143	21.0	11.358
5	42	15	0.789	34.799	32.474	617	37.0	23.388
6	7	1	0.167	4.162	1.833	11	8.0	2.828

12. *npubs*: Number of publications (based on Web of Science using all databases)
13. *h*: Hirsch's h-index (the number of publications cited at least h times each)
14. *mqPhD*: m quotient, which is h-index divided by academic age. Note that we define academic age as the number of years since conferral of the Ph.D. degree, which differs from the age used by Hirsch.
15. *hb*: Brown's hb-index (see manuscript for computation)
16. *citesperyrPhD*: Citations per year since Ph.D. (i.e., number of citations/academic age)
17. *nCites*: Total number of citations (based on Web of Science using all databases)
18. *m-index*: The median number of citations for papers in the h-core
19. *r-index*: The square root of the sum of citations for papers in the h-core

Next, read in the data file containing information about the potential new faculty member(s) you wish to evaluate. This file should be nearly identical in structure to the database above (one row per scientist). The file '*S2_Data.csv*' is provided as an example, containing three potential faculty members. This input file can potentially contain any number of individuals to evaluate. The rows of this file are named to uniquely identify the faculty candidates.

```
newdata <- read.csv('S2_Data.csv',header=TRUE)
rownames(newdata) <- c('candidate.x','candidate.y','candidate.z')
```

In the provided file, *candidate.x* represents the hypothetical candidate discussed in the paper. This individual is a male with a 25% research appointment, 15 years post-PhD, who works on wildlife diseases and ecology by developing quantitative tools.

```
newdata[,1:9]
```

	age.phd	Male	Re	health	genetics	social	applied	ecology	quant
candidate.x	15	1	25	1	0	0	0	1	1
candidate.y	15	0	60	0	1	0	0	0	0
candidate.z	20	1	5	0	0	0	0	1	0

candidate.x has the following values for the 8 response variables or metrics:

- Total publications = 30

- h-index = 10
- m-quotient (or rate of accumulation of h) is 10/15 = 0.67 from PhD date
- hb-index = 21
- Citations per year since PhD date = 18
- Total number of citations = 270
- m-index = 17 = median number of cites for papers in h-core
- r-index = 15 = square root of sum of citations in h-core

```
newdata[,12:19]
```

	nPubs	h	mqPhD	hb	citesperyrPhD	nCites	mindex	rindex
candidate.x	30	10	0.670	21.000	18.000	270	17	15.000
candidate.y	53	15	1.364	34.313	54.364	598	26	21.471
candidate.z	29	16	0.800	52.000	77.600	1552	50	38.756

Fit Regression Models

The faculty database is split into independent variables (X) and response variables (Y), and the appropriate information for the potential new faculty members is appended to X and Y to create $X.new$ and $Y.new$. These appended rows are named according to the provided ID values.

```
X <- alldata[,1:11]
Y <- alldata[,12:19]

X.new<-as.matrix(rbind(X,newdata[,1:11]))
row.names(X.new) <- c(1:dim(alldata)[1],as.character(newdata[,1]))
Y.new<-as.matrix(rbind(Y,newdata[,12:19]))
row.names(Y.new) <- c(1:dim(alldata)[1],as.character(newdata[,1]))
```

A quadratic term for *age.phd* is computed (*age.phd* is centered and squared):

```
age.2n <- scale(X.new[,1],center=TRUE,scale=FALSE)
age.2n <- age.2n^2
```

Finally, a series of unique generalized linear models (one per response variable or metric) are fit. Best fit models for each response variable were chosen as described in Swihart et al. (*in press*).

```
models <- list()
models$np.best <- glm.nb(Y.new[,1]~X.new[,1]+age.2n+X.new[,2:11])
models$h.best <- glm.nb(Y.new[,2]~X.new[,1]+age.2n+X.new[,3:11])
models$mq.best <- glm(Y.new[,3]~log(X.new[,1]+.5)+age.2n+X.new[,3:11])
models$hb.best <- glm.nb(round(Y.new[,4])~X.new[,1]+age.2n+X.new[,3:11])
models$c.best <- glm.nb(round(Y.new[,5])~X.new[,1]+X.new[,3:11])
models$nc.best <- glm.nb(Y.new[,6]~X.new[,1]+age.2n+X.new[,3:11])
models$m.best <- glm.nb(round(Y.new[,7])~X.new[,1]+age.2n+X.new[,3]+X.new[,4:11])
models$r.best <- glm.nb(round(Y.new[,8])~X.new[,1]+age.2n+X.new[,3]+X.new[,4:11])
```

Calculate Standardized Deviance Residuals

After all models are fit, standardized deviance residuals are calculated for each model and stored in the *stdres* object.

```
stdres <- sapply(models,rstandard)
```

We then calculate a series of summary statistics (mean, range, and 95% confidence interval) across all 8 metrics for each of the faculty included. These values will be used later.

```
r.mean <- rowMeans(stdres)
r.min <- apply(stdres,1,min)
r.max <- apply(stdres,1,max)
r.ci <- 1.96*apply(stdres,1,sd)/sqrt(dim(Y.new)[2])
```

A matrix *set* is defined to store quantile values for each faculty × metric combination for the full dataset. Summary statistics (mean, SD, and range) for each faculty calculated across the 8 metrics are stored in *qstat*.

```
set <- matrix(data=NA,nrow=dim(Y.new)[1],ncol=dim(Y.new)[2])
  for(i in 1:dim(Y.new)[1]){
  for(j in 1:dim(Y.new)[2]){
    set[i,j] <- sum(stdres[,j] <= stdres[i,j])/dim(Y.new)[1]
  }
  }

qstat <- matrix(data=NA,nrow=dim(Y.new)[1],4)
  for(i in 1:dim(Y.new)[1]){
  qstat[i,1] <- mean(set[i,])
  qstat[i,2] <- sd(set[i,])
  qstat[i,3] <- min(set[i,])
  qstat[i,4] <- max(set[i,])
  }

colnames(qstat) <- c('Mean','SD','Min','Max')
rownames(qstat) <- c(1:dim(alldata)[1],rownames(newdata))
```

```
head(qstat)
```

	Mean	SD	Min	Max
1	0.176	0.123	0.098	0.468
2	0.116	0.064	0.055	0.255
3	0.606	0.136	0.423	0.852
4	0.347	0.101	0.277	0.584
5	0.467	0.129	0.373	0.770
6	0.036	0.034	0.009	0.116

Thus, for example, the first faculty member in the database has an average quantile value of 0.176 with a standard deviation of 0.123 across the 8 metrics.

Evaluation of Individual Faculty

In this section, we focus on the set of faculty members being evaluated based on the existing database (that is, the individuals contained in the '*Inputfaculty.csv*' file, 3 individuals in total). Hereafter, we refer to these 3 individuals as 'candidates'.

First, using *stdres*, we obtain quantile values for each candidate × metric combination assuming residuals come from a standard normal distribution:

```
first <- dim(alldata)[1] + 1 # index of first candidate
last <- dim(alldata)[1] + dim(newdata)[1] # index of last candidate
quantile.normal <- round(t(apply(stdres[first:last,],1,pnorm)),3)
colnames(quantile.normal) <- colnames(Y.new)
row.names(quantile.normal) <- row.names(newdata)
```

```
quantile.normal
```

	nPubs	h	mqPhD	hb	citesperyrPhD	nCites	mindex	rindex
candidate.x	0.155	0.146	0.169	0.079	0.068	0.085	0.070	0.088
candidate.y	0.487	0.387	0.596	0.289	0.283	0.223	0.246	0.247
candidate.z	0.222	0.653	0.610	0.821	0.831	0.810	0.852	0.881

Using the summary statistics calculated earlier (mean, 95% confidence interval, range) for each candidate across residuals from all metrics we calculate a quantile value for each candidate × statistic combination (still assuming a standard normal distribution for residuals):

```
summary.normal <- matrix(NA,nrow=dim(newdata)[1],ncol=5)
colnames(summary.normal) <- c('Mean','2.5%','97.5','Min','Max')
rownames(summary.normal) <- rownames(newdata)
summary.normal[,1] <- pnorm(r.mean[first:last])
summary.normal[,2] <- pnorm(r.mean[first:last] - r.ci[first:last])
summary.normal[,3] <- pnorm(r.mean[first:last] + r.ci[first:last])
summary.normal[,4] <- pnorm(r.min[first:last])
summary.normal[,5] <- pnorm(r.max[first:last])
summary.normal <- round(summary.normal,3)
```

```
summary.normal
```

	Mean	2.5%	97.5	Min	Max
candidate.x	0.103	0.078	0.133	0.068	0.169
candidate.y	0.338	0.252	0.433	0.223	0.596
candidate.z	0.729	0.567	0.854	0.222	0.881

Next, we take an alternative approach to calculation of quantiles We calculate quantile values for each candidate × metric combination in relation to the larger faculty database (instead of assuming a normal distribution).

```
quantile.database <- round(set[first:last,],3)
colnames(quantile.database) <- colnames(Y.new)
row.names(quantile.database) <- row.names(newdata)
```

```
quantile.database
```

	nPubs	h	mqPhD	hb	citesperyrPhD	nCites	mindex	rindex
candidate.x	0.177	0.159	0.091	0.093	0.102	0.125	0.086	0.109
candidate.y	0.607	0.436	0.689	0.311	0.384	0.300	0.289	0.277
candidate.z	0.289	0.723	0.714	0.866	0.877	0.882	0.893	0.902

Next, for each candidate, we calculate a set of summary statistics (mean, 95% confidence interval, and range) across the 8 metrics from the table above.

```
summary.database <- matrix(NA,nrow=dim(newdata)[1],ncol=5)
colnames(summary.database) <- c('Mean','2.5%','97.5%','Min','Max')
row.names(summary.database) <- rownames(newdata)
summary.database[,1] <- qstat[first:last,1]
rp.ci <- 1.96*qstat[first:last,2]/sqrt(dim(Y.new)[2])
summary.database[,2] <- summary.database[,1] - rp.ci
summary.database[,3] <- summary.database[,1] + rp.ci
summary.database[,4] <- qstat[first:last,3]
summary.database[,5] <- qstat[first:last,4]
summary.database <- round(summary.database,3)
```

```
summary.database
```

	Mean	2.5%	97.5%	Min	Max
candidate.x	0.118	0.095	0.141	0.086	0.177
candidate.y	0.412	0.303	0.520	0.277	0.689
candidate.z	0.768	0.624	0.912	0.289	0.902

Multivariate Performance Benchmarking

Here we demonstrate a factor analysis approach for benchmarking the performance of individual faculty candidates. First, conduct the factor analysis and examine the results:

```
fa <- factanal(stdres,factors=2,scores='reg',rotation="varimax")
```

Using the results from the factor analysis, plot convex hulls for 100, 95, 90, 75, and 50% of the factor scores. The locations of the three candidates along the factor axes are highlighted.

```
p100 <- plothulls(fa$scores,fraction=1.0,main="",pch=20,cex=.65,lwd=2,col="gray",
                  xlab="Factor 1",ylab="Factor 2")
p95 <- plothulls(fa$scores,fraction=0.95,add=TRUE,lwd=2,main="",pch=20,cex=.65,col="gray")
p90 <- plothulls(fa$scores,fraction=0.9,add=TRUE,lwd=2,main="",pch=20,cex=.65,col="gray")
```

```
p75 <- plothulls(fa$scores,fraction=0.75,add=TRUE,lwd=2,main="",pch=20,cex=.65,col="gray")
p50 <- plothulls(fa$scores,fraction=0.5,add=TRUE,lwd=2,main="",pch=20,cex=.65,col="gray")
abline(h=0,lty=2,lw=2)
abline(v=0,lty=2,lw=2)
abline(a=0,b=1,lty=3,lw=2,col="gray")

points(x=fa$scores[first,1],y=fa$scores[first,2],col="red",pch=16,cex=1.5,lwd=2)
points(x=fa$scores[first+1,1],y=fa$scores[first+1,2],col="blue",pch=16,cex=1.5,lwd=2)
points(x=fa$scores[first+2,1],y=fa$scores[first+2,2],col="green",pch=16,cex=1.5,lwd=2)
legend('topright',legend=c('candidate.x','candidate.y','candidate.z'),
       pch=16,cex=1,col=c('red','blue','green'))
legend(1,5.25,legend="I",bty="n")
legend(-3.5,5.25,legend="II",bty="n")
legend(-3.5,-2,legend="III",bty="n")
legend(2.4,-2,legend="IV",bty="n")
```

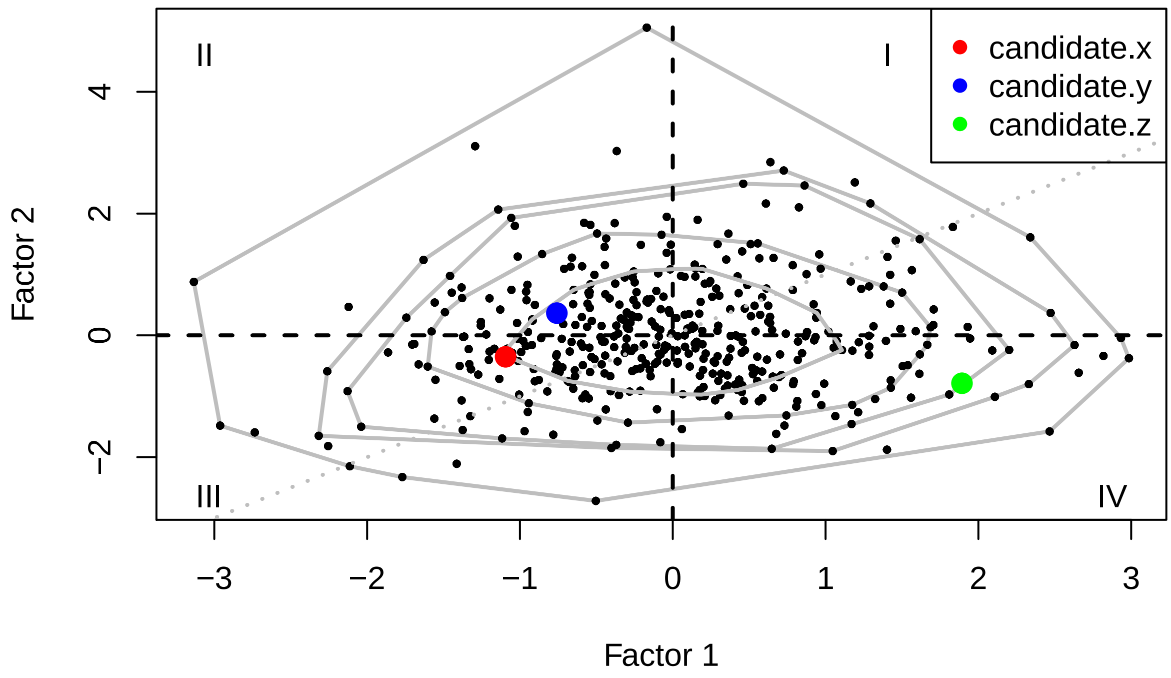

Next, calculate the quantiles for the factor scores (considered separately) of the candidates relative to the faculty database:

```
q <- matrix(data=NA,nrow=dim(Y.new)[1],ncol=dim(fa$scores)[2])
for(i in 1:dim(Y.new)[1]){
  for(j in 1:dim(fa$scores)[2]){
    q[i,j] <- sum(fa$scores[,j] <= fa$scores[i,j])/(dim(Y.new)[1])
  }
}
```

```r
factor.quantiles <- round(q[first:last,],3)
colnames(factor.quantiles) <- c('Factor 1','Factor 2')
row.names(factor.quantiles) <- row.names(newdata)
```

```r
factor.quantiles
```

	Factor 1	Factor 2
candidate.x	0.116	0.361
candidate.y	0.202	0.691
candidate.z	0.968	0.186

Now we obtain a single score for each candidate, by averaging the factor scores together while weighting the scores by the corresponding eigenvalues (i.e., proportional to the amount of variation each factor explains). We then rank each candidate (out of 440 total faculty) according to the averaged factor score.

```r
lam <- colSums(fa$loading*fa$loading)/dim(fa$loading)[1] #calculate prop var explained
f <- matrix(data=NA,nrow=2,ncol=1)
f[1] <- lam[1]/sum(lam)  #weight for factor 1
f[2]<- lam[2]/sum(lam)   #weight for factor 2
s.w <- fa$scores%*%f
factor.average <- matrix(NA,nrow=dim(newdata)[1],ncol=2)
colnames(factor.average) <- c('Score','Rank')
row.names(factor.average) <- row.names(newdata)

factor.average[,1] <- round(s.w[first:last],3)

sw.rank <- rank(s.w) #rank factor scores

factor.average[,2] <- round(sw.rank[first:last])
```

```r
factor.average
```

	Score	Rank
candidate.x	-0.807	48
candidate.y	-0.323	129
candidate.z	0.855	388
