# Supplementary material for: Performance Benchmarks for Scholarly Metrics Associated with Fisheries and Wildlife Faculty
Source: PLoS One. 2016 May 6;11(5):e0155097. doi: 10.1371/journal.pone.0155097 (PMC4859475; doi:10.1371/journal.pone.0155097)
Supplement: S1 Table — (DOCX) [file pone.0155097.s004.docx]

**S1 Table. Correlations of standardized deviance residuals from 8 models for 437 faculty in fisheries and wildlife.**
