# Supplementary material for: Performance Benchmarks for Scholarly Metrics Associated with Fisheries and Wildlife Faculty
Source: PLoS One. 2016 May 6;11(5):e0155097. doi: 10.1371/journal.pone.0155097 (PMC4859475; doi:10.1371/journal.pone.0155097)
Supplement: S2 Table — Normalization was accomplished by dividing values by the corresponding citations per article for mathematics. (DOCX) [file pone.0155097.s005.docx]

**S2 Table. Average annual normalized citations per article for JCR categories and associated fisheries and wildlife sub-disciplines as grouped in Table 2.**
